# Recycling of Carbon Fiber Reinforced Composite Polymers—Review—Part 2: Recovery and Application of Recycled Carbon Fibers

**DOI:** 10.3390/polym12123003

**Published:** 2020-12-16

**Authors:** Andrzej K. Bledzki, Holger Seidlitz, Jonas Krenz, Krzysztof Goracy, Magdalena Urbaniak, Janina J. Rösch

**Affiliations:** 1 Faculty of Mechanical Engineering and Mechatronics, West Pomeranian University of Technology in Szczecin, 70-310 Szczecin, Poland; magdalena.urbaniak@zut.edu.pl; 2 Chair of Polymer-Based Lightweight Design, Brandenburg University of Technology Cottbus—Senftenberg, 03046 Cottbus, Germany; holger.seidlitz@b-tu.de (H.S.); Jonas.Krenz@b-tu.de (J.K.); Janina.JR.Roesch@bmw.de (J.J.R.); 3 Polymeric Materials and Composites PYCO, Fraunhofer IAP, 14513 Teltow, Germany; 4 Faculty of Chemical Technology and Engineering, West Pomeranian University of Technology in Szczecin, 70-322 Szczecin, Poland; krzysztof.goracy@zut.edu.pl; 5 Digitalization Production, BMW Group, 80788 Munich, Germany

**Keywords:** polymer composites, recycled carbon fibers, recycling, automotive industry, railways, aviation, wind turbine

## Abstract

The paper presents some examples of new technological solutions for the recovery and re-use of recycled carbon fiber in automotive and railway industries, as well as in aviation and wind turbine constructions. The new technologies of fiber recovery that are described can enable the mass-scale use of recycled carbon fiber in the future.

## 1. Introduction

The first part of the paper [1] focused on the current volume of international production and on global markets of carbon fiber reinforced polymer composites, also regarding the potential development trends. Examples were provided on how to effectively recycle carbon fiber reinforced polymer composites. Legally binding legislation in the EU on polymer composite recycling was also presented.

The second part of this paper provides examples of the recovery and re-use of recycled carbon fiber in the automotive and railway industries, as well as in aviation and wind turbine construction.

## 2. Automotive Industry

Much hope lies in the automotive industry perceived as the customer. Seventeen million vehicles are built annually in Europe. Each of them requires an average of 120 kg of plastics, with 20% being composites [2]. Recycled carbon fiber (rCF) composites can provide a good surface and low weight. The drawbacks of rCF composites include processing time and cost [3]. New trends in the automotive sector focus on reducing pollution emissions, new engines, autonomous and electric cars and vehicle data transmission. The whole sector is being restructured and new materials are being selected. Car designers will surely consider the advantages of polymer composites, including rCF composites.

Some rCF producers complain that car manufacturers are reluctant to take the risk—there must always be someone to be the first to implement new materials. However, the same producers admit that widespread use of rCF in cars would soon exhaust the potential of companies that recover fibers. That is why for the time being they are satisfied to take part in small-scale projects, trying to enter the automotive industry in the same way as polymer composites, i.e., starting from less important components and then moving on to pressure tanks [3]. BMW Group is a good example of users of rCF in the automotive industry.

Carbon fiber reinforced polymer (CFRP) is increasingly used in several industry sectors. The global demand for CFRP is forecast to reach 121,015 tons in 2020, up by 11% (compared to 2019) [4]. The installation of, e.g., electric motors in the vehicle increases the total vehicle weight, so that solutions for breaking the weight spiral must be found. CFRP is mainly used in the automotive industry in exterior components. In recent years, the focus has increasingly shifted to load-bearing components in vehicles [5]. When looking at the percent weight distribution of the vehicle, it can be observed that the car body accounts for a large proportion of the total vehicle weight (40%) [5,6]. Therefore, investigations to reduce the weight of body structures are under way. After the use of CFRP in mass applications in automotive engineering, development is increasingly moving towards hybrid construction methods. Compared to metallic materials, CFRPs have directional material properties and differ in fiber orientation [7,8].

There are various applications for the use of recycled composite material within the automotive industry. The series production of the BMW i3 and i8 started in 2013. Before this, BMW Group and Airbus agreed on cooperation regarding the recycling of carbon fibers. This cooperation is important for both companies regarding the development of carbon recycling and re-use methods [9]. Another important cooperation regarding the development of methods is the Joint Venture SGL Automotive Carbon Fibers LLC in Moses Lake, Washington State (USA) with BMW Group and SGL Group [9].

**Figure 1:**
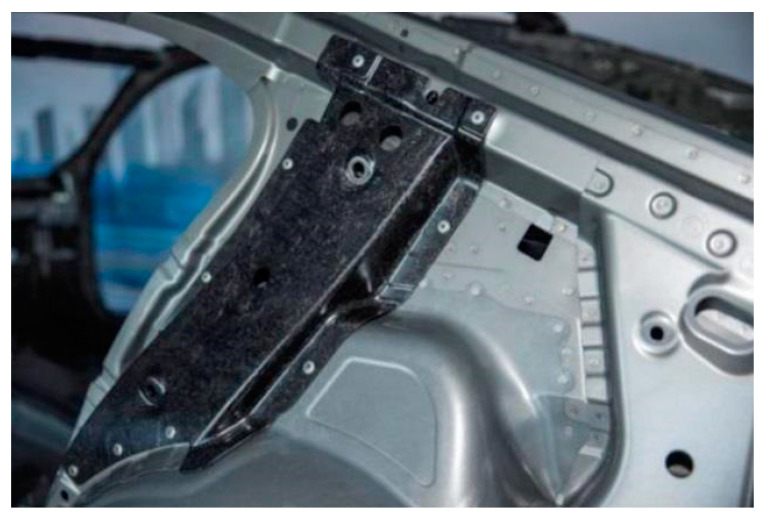
C-pillar within BMW 7 series from sheet molding compound (SMC).

The shredding produces short fibers. These can then be re-used for the production of non-woven material and mats, and they are the most widespread semi-finished products, which are used especially for sheet molding compound (SMC) semi-finished products [10]. BMW Group is using recycled carbon fiber for the production of the reinforcement of the C-pillar with SMC (sheet molding compound) material (Figure 1) [11]. Toyota is using Mitsubishi Rayon’s (MRC) SMC material for the production of the hatch door frame [11]. Furthermore, non-wovens are used as cover layers in component production to increase surface quality, especially for exterior parts [10].

**Figure 2:**
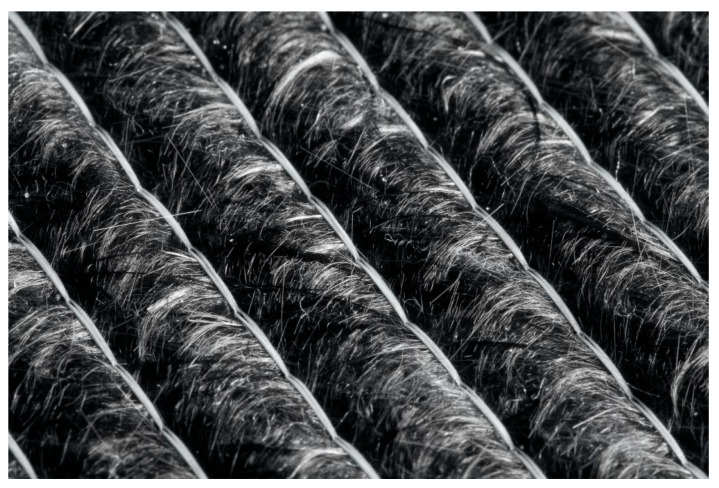
Carbon fiber recycling material for use in the BMW i3, e.g., the roof of the BMW i.

Another example from BMW Group’s recycling concept is the roof of the BMW i3 (Figure 2). It consists entirely of a carbon fiber blend. This material, which has not yet come into contact with resin, is part of the recycling within the pre-processing stage. Regarding the BMW i3, up to 95% of the materials used in its construction are recyclable, and carbon fibers can be re-introduced into the production process [12,13]. Figure 2 gives a close-up of the recycled material used in the BMW i3 roof. With this approach the waste material during the preforming process can be re-used during production and even improve the resin transfer process. Due to the lower fiber volume of the non-woven material, it can improve the resin transfer and shorten the injection process.

According to the AVK-Report (Arbaitsgemeinschaft Verstearktekunststoffe, Federation of Reinforced Plastics) dry carbon fibers and prepregs, impregnated with resin but not yet cured, represent the largest waste. “For this, our major topic is plastic injection moulding with recyclate”, Brúch (BMW Group—company speaker). One example is the center console of the current MINI, which is manufactured with granulate from chopped CFRP parts [14].

Within the automotive industry, companies use service providers for the recycling process. CFK Valley Stade Recycling GmbH & Co. KG, a certified waste management company, offers sustainable recycling of carbon fiber production waste and end-of-life components. The company offers recycling concepts and has development contracts with, i.e., Airbus or Bugatti. First, the dry fiber, prepregs and cured CFRP parts are sorted according to fiber length and processing stage. Dry fibers are chopped. These chopped fibers can have an average fiber length of 6, 12, 20, 60 or 80 mm with a tensile strength of >3500 MPa and tensile modulus of >230 GPa. If the dry fibers are chopped, they can reach fiber lengths of 80–500 μm with a diameter of around 6 μm. For cured CFRP parts, the pure carbon fibers are then completely recovered by means of thermal treatment. Secondly, the refinement and final process begins. CFK Stade Recycling GmbH & Co. KG will use carboNXT “chopped” and carboNXT “milled” processes [15].

The company carboNXT offers various recycled materials, such as milled or granulated carbon fibers as well as high-dose carbon fibers. Other possible materials are carbon fiber reinforced thermoplastics and duroplastic molding compounds. Non-woven and carbon fiber papers are also recyclates. The materials can be used as raw material for the production of the following components within the automotive industry [16]:

- engine covers,
- anti-corrosion covers,
- fiber-reinforced plastic surfaces,
- interior parts, i.e., the cover of navigation covers,
- fiber-reinforced front and rear bumpers, and
- abrasion-proof chain tensioners.

The use of different recycling methods and the outcoming material needs to be chosen according to the component requirements and production technology. Milled fiber, for example, can be used as filler material to improve mechanical and electrical properties for plastic housings or covers. Milled fibers can be used for SMC compounds or non-woven mats. rCF SMC is suitable for constructions where high mechanical strength is required [15].

Due to the use of recycled carbon fiber, which costs 20–40% less than virgin carbon fibers, new possibilities are available [16] for use within the automotive sector.

## 3. Aviation Industry

The ideal situation would be to recover carbon fiber from industrial waste generated, for example, while cutting out preforms from fabrics with/without a thermoplastic matrix, without losing their properties [17]. The aviation industry, which generates large amounts of waste, could be a big source of recycled materials. A new tendency in aviation is to use thermoplastic composites, which can facilitate recycling and rCF use. Many thermoplastic composite materials are already applied in airplane building, meaning the technology and equipment are both available [3].

The Dutch Fokker, Toray, and other companies specializing in design, processing and recycling took part in a project attempting to prove that it is possible to obtain high-quality thermoplastic composites from rCF, thus reducing the environmental burden and cost. The project also aimed to analyze costs throughout the whole life cycle of the material, and it showed that rCF use is economically profitable [3]. Fokker is also examining whether or not recycling technology can be part of a new program of building new generation hulls. Fokker argues that new materials can set them free from design and technological constraints resulting from the use of laminate and metal materials.

## 4. Carbon Fiber Recovery from Wind Turbines

Most wind power plants operate with three blades. Blades account only for approximately 4% of the total weight of the structure (for 1–3 MW turbines). Composites (mainly glass fiber and thermosetting resin) make up roughly 40% of blade weight. Blade production generates about 10% of waste, i.e., approximately 1200 tons of composite material annually [2]. However, the problem is much greater if you take into account that the average exploitation time of a wind turbine is 20–25 years. Within the next 20 years, the estimated amount of composite waste from turbines will exceed 1 million tons [2]. Additionally, because of rising costs and falling profits, the number of turbines will continue to decrease. An estimated 2000 turbines will be retired from use annually. Between 80 and 90% of all materials used for wind power plants are recycled, except for blades and nacelles [18].

BWE (Bundesverband Wind Energie, Federal Association of Wind Energy, Berlin, Germany) has officially claimed that the situation is not that dramatic. This is despite the fact that the volume of blade waste material will increase from 10,000 tons to 40,000 tons in 2040. Because of high loads, only composite materials are going to be used, mainly epoxy/glass composites. Current methods of recycling rotors and nacelles involve their combustion in cement plants. Rotorblades are dismantled, separated in GFRP (glass fiber reinforced polymer) and CFRP (carbon fiber reinforced polymer) parts, shredded in an approximate diameter of 200 mm flakes and shredded again through a turbo-crusher, to a diameter of 35–50 mm at the NEOCOMP Facility. Within the shredding process, metal parts are separated with the help of magnetic fields. It is not possible to recycle the CFRP parts in cement plants; hence, all CFRP parts need to be manually cut out before shredding. The turbo crusher is loaded with the wet reject waste of the paper recycling process (60%_m_). This waste and GFRP is homogenized and stored for burning in cement plants. The use of GFRP in cement plants follows two recycling strategies. Glass fibers are substituted for sand, and matrix material is used to heat up the recycling process in the rotary kiln [19]. Research is underway to examine the use of high-temperature hydrolysis (over 1100 °C, at 30% humidity) with gas and glass recovery that can be further used as non-structural glass [18].

CF (carbon fiber) recycling has a number of drawbacks, often mentioned by wind turbine manufacturers. Fibers become shorter in each subsequent recycling cycle, which significantly reduces their application in this technological sector. Shredding can also produce powder that is harmful to humans and the environment. CF recovered from turbines is always contaminated with GF (glass fiber), which makes their re-use difficult and is not economically viable. The combustion of CF composites is not a completely environmentally sustainable solution. Additionally, since CF can conduct electricity, it may lead to short circuiting or even to fires during filtering installations if fibers have not burned out completely [18].

There is no method of recycling CF composites from wind turbines on a mass-scale. Therefore, some specialists recommend to avoid, whenever possible, the use of CF in the production of turbine blades [18].

As the wind industry grows and blade waste levels climb into the tens and hundreds of thousands of tons, and beyond, a better end-of-life (EoL) solution is needed. It was reported lately that the University of Tennessee (USA) received $1.1 million in funding from the Department of Energy’s (DOE) Small Business Technology Transfer (STTR) program and Wind Energy Technologies Office for the large-scale recycling of wind turbine blades into new recycled composites. Over the next two years the University of Tennessee will be collaborating with some specialized companies to develop a pilot-scale recycling system that will serve as the basis for the eventual deployment of a full-scale commercial wind blade waste processing plant [20].

## 5. Railway Industry

ELG Carbon Fibre Ltd. (ELG) materials have been used to produce a two-axel bogie for British Rail, and every railway wagon uses two such bogies. New fibers were added to rCF mats. The weight of a bogie was reduced by 60%, compared to a steel structure [21]. It is assumed that 1 kg of CF composite can replace 3 kg of steel in structural components. Given that the weight of a typical steel bogie is 1500 kg, substantial cost reduction can be achieved in wagon exploitation and travel cost. Taking into account railway mileage and the cost of electricity involving inflation and maintenance, savings of £10 per kilogram of reduced weight for commuter trains and approximately £100 for high-speed trains can be achieved. This technology also substantially reduces CO_2_ emissions during train operation [22].

The development of the new bogie shows what kind of challenges need to be faced by companies and institutions that introduce rCF into the market. The concept, design, tests, material qualification, development of production methods and ensuring that the new material met the standards of connecting with other components took three years. New CFs were applied in places that required reinforcement or strengthening [22].

The use of rCF in already existing products imposed a number of limitations, e.g., the geometry of connection with the wagon frame. This is why the bogie prototype does not always optimally take advantage of the composite’s properties of stiffness and strength [22]. The new frame is 64% lighter than the steel frame, but the whole prototype is “only” 36% lighter. This discrepancy is due to the weight of paint and the steel elements necessary to connect the bogie with the rest of the vehicle.

Previously, the use of CF in bogies was limited by the price of fibers. Currently, the application of rCF makes it profitable given the involvement of several companies, its re-design and fatigue tests. Having assumed that the UK market needs approximately 36,000 bogies for passenger wagons within the next 10 years, the project seems to be profitable [22]. Other beneficiaries interested in wagon weight reduction are companies responsible for rail maintenance and repair, and those that monitor railway use.

The production of the new bogie helped to persuade the very conservative railway industry that composite materials, particularly those involving rCF, can be effectively used for structures working under load and not only for non-structural components of wagon interiors. The consortium had to do research and compare the mechanical, fatigue and strength properties of new materials with their steel counterparts. Fatigue load resistance and flammability in particular had to meet railway requirements. In the case of flammability tests (fire spread, heat release, smoke density), the whole procedure needed revising, i.e., whether or not the tests should apply to the surface of the new material alone or its whole volume.

After the new bogie model has been introduced, the consortium is planning to further optimize the design by changing the approach of design engineers to persuade them to take advantage of composite materials, their possibilities and properties [22].

## 6. Recycled Carbon Fiber (rCF) for CFRP Mold Production

The advantage of using CFRP as a mold material is that it has a very similar thermal expansion to the cured CFRP part. Due to this property, when cooling down the parts in an autoclave or oven there is a very low tension between the mold and the part. In comparison to aluminum, steel or invar, CFRP shell-based molds are light and therefore have excellent handling properties. Because of these two reasons it is common to use CFRP tooling material. One solution is to use chopped thermoset prepreg tapes, which are randomly oriented in thick sheets [23].

**Figure 3:**
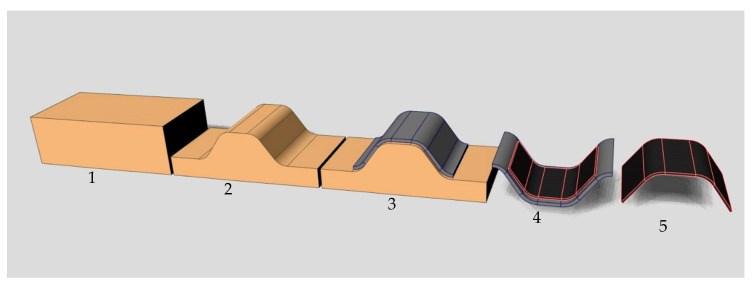
Manufacturing process of recycled carbon fiber (rCF)-based fiber-reinforced polymer (FRP) molds: (**1**) master mold is made of polyurethane cuboid; (**2**) milled master mold; (**3**) draping and infusion of rCF-Mats; (**4**) manufacturing of FRP-parts; (**5**) final and removed FRP part.

Another cost-effective alternative would be to use non-woven rCF mats and infuse those mats on Computerized Numerical Control (CNC)-machined master-molds. On account of the low costs of rCF mats, CFRP molds could close the recycling loop. Cost-effective rCF-Mats from ELG up to 500 g/m^2^ areal density and width up to 2.6 m are already in market [24]. Figure 3 illustrates the manufacturing process of rCF-based FRP-molds.

The first practical application of these rCF-Mats could be seen in boat hull shells of the INEOS Team UK. Driven by the sustainable construction requirement of the America’s Cup Class Rules for 2021, rCF is used by the sailing team. ELG worked together with the INEOS Team, UK, to use rCF-Mats for the AC75 boat in the 2021 America’s Cup. Non-woven materials are used in the production of two cradles to support the boat during transit. The material is also used for the production of the hull, deck molds and tooling [25,26,27].

In addition to the classic production of molds in the marine sector using master molds and the molding of shells via infusion, large-scale additive manufacturing (LSAM) is also being used already as a cost-effective short-term process in the aviation field. In the above-mentioned process, thermoplastic granulates filled with 20–25% milled carbon fibers are melted and extruded in layers around the final contour, using gantry systems or robots.

**Figure 4:**
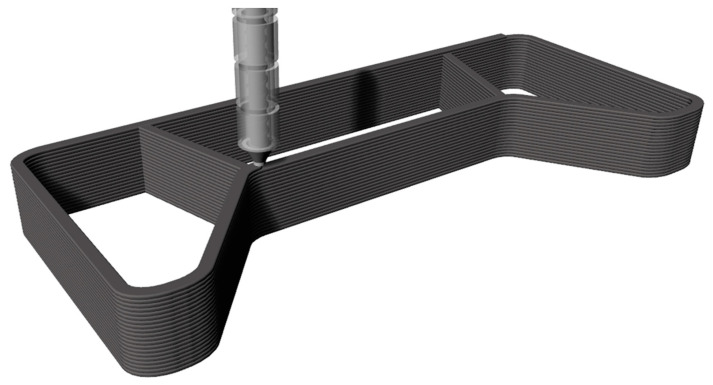
CNC extrusion process of thermoplastic granulate filled with carbon fibers.

Figure 4 shows the extruded mold and a simplified scheme of the extruder which is mounted on a gantry or robot system. The molten granulate is placed in lines in contour of the final FRP mold.

**Figure 5:**
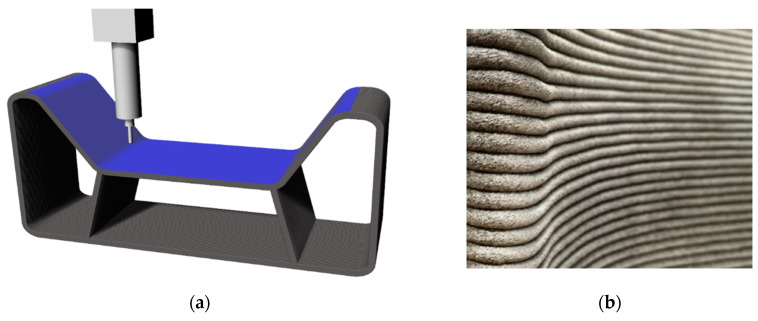
The cavity needs to be prepared in the following step: illustration of the fine finishing process (**a**); lines of extruded granulate before the finishing process to smoothen the surface (**b**).

The extrusion process is followed by a quick fine finishing process, which realizes the required position tolerance and the surface quality. Figure 5a presents the finishing process with a milling machine of the blue illustrated cavity, and Figure 5b shows a detailed photograph of an extruded contour, which needs to be finished, before using it for molding processes.

A popular example of LSAM technology is the production of a helicopter rotor blade mold, which is made from polyether sulfone (PESU) with 25% carbon fibers. The mold with the approximate dimension of 6 × 0.35 × 0.4 m was additively manufactured in just 3 h and 8 min. By using PESU it is possible to use the autoclave to cure composite parts up to 6 bar at 180 °C [28].

**Figure 6:**
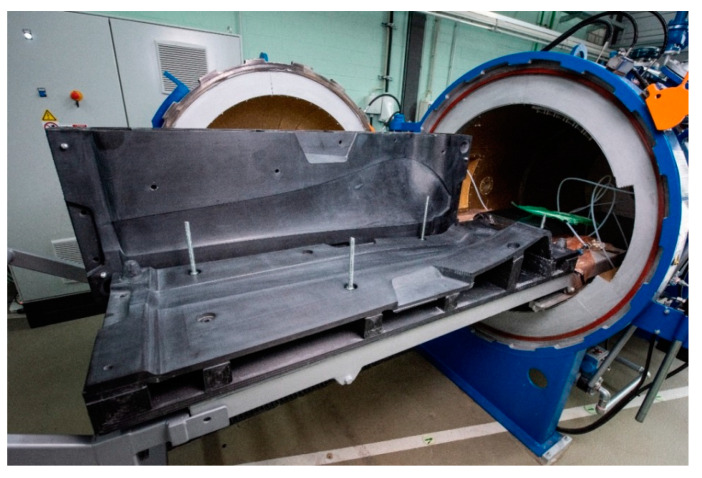
Direct rotorblade mold, made with LSAM technology.

Similar projects have been done in collaboration between Brandenburg University of Technology (BTU) Cottbus-Senftenberg and CNC Barcenas (Valdepeñas, Spain). Molds for small wind turbines were printed and fine finished with SABIC Material ABS-20CF and used for infusion process and autoclave curing. Figure 6 shows the final manufactured FRP mold.

The use of rCF in the above-mentioned process instead of virgin carbon fibers is obvious, since the high price of the granulate is primarily determined by the carbon fiber percentage. The reason for using CF is mainly to decrease the warping effect while cooling down the printed layers. Initial studies on the use of rCF in large-scale additive manufacturing processes are already being carried out by the OAK Ridge National Laboratory and are in the development stage at BTU Cottbus-Senftenberg [29].

## 7. Summary

It would seem that the future of CFRP recycling is optimistic. New technologies of fiber recovery have been developed, and according to the first estimates, fiber recovery is profitable. The first supply chains of materials to be recycled have been organized. The first technological solutions enabling mass-scale use of rCF are also in place.

However, CFRPs had a similar starting point, and yet their recycling in the global scale has not been solved so far. Do we again face a future of empty declarations, presentations and the production of niche products?
